# Arbuscular mycorrhizal fungi enhance disease resistance of *Salvia miltiorrhiza* to Fusarium wilt

**DOI:** 10.3389/fpls.2022.975558

**Published:** 2022-12-01

**Authors:** Chunjuan Pu, Yang Ge, Guang Yang, Han Zheng, Wei Guan, Zhi Chao, Ye Shen, Sha Liu, Meilan Chen, Luqi Huang

**Affiliations:** ^1^ School of Pharmacy, Nanjing University of Chinese Medicine, Nanjing, China; ^2^ State Key Laboratory of Dao-di Herbs, National Resource Center for Chinese Materia Medica, China Academy of Chinese Medical Sciences, Beijing, China; ^3^ State Key Laboratory for Biology of Plant Diseases and Insert Pests, Institute of Plant Protection, Chinese Academy of Agricultural Sciences, Beijing, China; ^4^ School of Traditional Chinese Medicine, Southern Medical University, Guangzhou, China

**Keywords:** arbuscular mycorrhizal fungi, disease resistance, *Fusarium oxysporum*, Fusarium wilt, *Salvia miltiorrhiza*

## Abstract

*Salvia miltiorrhiza* Bunge (Danshen in Chinese) is vulnerable to Fusarium wilt, which severely affects the quality of the crude drug. Mycorrhizal colonization enhances resistance to fungal pathogens in many plant species. In this study, pre-inoculation of *S. miltiorrhiza* with the arbuscular mycorrhizal fungi (AMF) *Glomus versiforme* significantly alleviated Fusarium wilt caused by *Fusarium oxysporum*. Mycorrhizal colonization protected *S. miltiorrhiza* from pathogen infection, thereby preventing a loss of biomass and photosynthesis. There were greater defense responses induced by pathogen infection in AMF pre-inoculated plants than those in non-treated plants. AMF pre-inoculation resulted in systemic responses upon pathogen inoculation, including significant increases in the protein content and activities of phenylalanine ammonia-lyase (PAL), chitinase, and β-1,3-glucanase in *S. miltiorrhiza* roots. In addition, mycorrhizal pre-inoculation caused upregulation of defense-related genes, and jasmonic acid (JA) and salicylic acid (SA) signaling pathway genes after pathogen infection. The above findings indicate that mycorrhizal colonization enhances *S. miltiorrhiza* resistance against *F. oxysporum* infection by enhancing photosynthesis, root structure, and inducing the expression of defense enzymes and defense-related genes on the other hand.

## Introduction

*Salvia miltiorrhiza* Bunge, a diploid species belonging to the family Lamiaceae (Li et al., 2017), is an industrially important medicinal plant widely used for treatment of coronary and cerebrovascular diseases (Chen et al., 2018; Liu et al., 2018; Shi et al., 2018). With increasing demand for *S. miltiorrhiza* in domestic and international markets, the planting area has expanded in China (Shi et al., 2018). *S. miltiorrhiza* is mostly cultivated with large-scale continuous planting, but Fusarium wilt caused by *Fusarium oxysporum* is a major threat (Zhang et al., 2016). Fusarium wilt is a fast-spreading epidemic disease that causes severe damage to the quality and productivity of *S. miltiorrhiza*, similar to the damage experienced by crops such as cucumber, chickpeas, banana, and cotton (de Lamo and Takken, 2020; Ankati et al., 2021; Qi et al., 2022). It is estimated that up to 70% of continuously cropped *S. miltiorrhiza* is affected (Yang et al., 2013). Synthetic fungicides are commonly used to control Fusarium wilt, however their use causes environmental pollution and threatens human health (Neeraj and Singh, 2011). Therefore, there is an urgent need for the identification of new biological control methods to suppress Fusarium wilt in the agricultural production of *S. miltiorrhiza* (Hammad and El-Mohandes, 1999).

In response to fungus pathogens, plants have evolved a series of complex strategies to protect themselves from damage (Song et al., 2015). Symbisis between plant root systems and arbuscular mycorrhizal fungi (AMF) can be exploited for crop disease management (Ajit et al., 2017). Arbuscular mycorrhizal symbiosis can enhance plant resistance against various pathogens such as *Alternaria* spp., *Rhizoctonia*, *Fusarium*, *Verticillium*, and *Thielaviopsis* (Nair et al., 2015; Mustafa et al., 2017). The protective effects may result from a combination of diverse mechanisms (Dey and Ghosh, 2022). AMF induced plant defense response plays an important role in plant disease resistance (Jung et al., 2012). The defense responses of plants can be pre-axisting and induced (Xu et al., 2022). Plant physical structures and phytochemicals provide basic defense against fungal pathogens (Robert-Seilaniantz et al., 2011; Bellincampi et al., 2014; Ziv et al., 2018). After recognition of fungal pathogens, defense signaling is activated, leading to induction of immunity, local defense responses, and systemic defense signaling (Tian et al., 2016). Mycorrhiza-induced resistance is characterized by induction of root cell wall thickening, accumulation of phytoalexins, induced expression of plant defense genes, and stimulation of plant defense enzymes such as PAL, chitinase, and β-1,3-glucanase (Song et al., 2015; Eke et al., 2016; Bai et al., 2018).

Pathogen infection can reduce plant photosynthesis and damage the root system of plants (Dong et al., 2016). Reduced photosynthesis prevents plants from obtaining carbon nutrients, and root damage limits the absorption of nutrients and water (Serrano et al., 2016). In previous research, we observed that AMF increases photosynthesis and improves the root system of plants (Chen et al., 2017b). Therefore, we investigated if AMF can alleviate the photosynthesis and the root structure damage leading to reduced yield of *S. miltiorrhiza* caused by pathogen infection.

Previously, we found that arbuscular mycorrhizal symbiosis decreases the disease incidence of continuously cropped *S. miltiorrhiza* by nearly 75% (Yang et al., 2013). However, there is little known about the response of AMF-inoculated *S. miltiorrhiza* to *F. oxysporum* infection and mycorrhizal-induced defense mechanisms are poorly understood. In this study, we investigated the mechanisms of defense response in *S. miltiorrhiza* against *F. oxysporum* infection induced by pre-inoculation with AMF from two perspectives: the photosynthesis and root structure, and changes in expression of defense-related genes.

## Materials and methods

### Plant materials and fungal strains

*S. miltiorrhiza* seeds were collected from a planting base located in Laiwu, Shandong Province in North China (36°20’ N, 117°41’ E). The authors identified the seedlings as *S. miltiorrhiza* Bunge.

The AMF *G. versiforme* was originally provided by Professor Honggang Wang (Chinese Academy of Agricultural Sciences). and was propagated using *Sorghum bicolor* as the host. The spores, hyphae, colonized roots, and substrates were collected as AMF inocula. The AMF inocula was identified as *G. versiforme* following Wang et al. (2016) described (**Figure S1**).

The pathogen was isolated from roots of diseased *S. miltiorrhiza* that showed symptoms of Fusarium wilt and identified as *F. oxysporum* (Yang et al., 2013). The pathogen was cultured for five days in Armstrong Fusarium Medium Base (20.0 g glucose, 0.2 mg FeSO_4_, 1.6 g KCl, 0.4 g MgSO_4_·7H_2_O, 5.9 g Ca(NO_3_)_2_, 0.2 mg ZnSO_4_, 1.1 g KH_2_PO_4_, and 0.2 mg MnSO_4_ per liter, pH 7.0) at 28°C in darkness and on a shaker at 150 rpm. Three layers of sterile gauze were used to filtrate mycelia and the suspension concentration was 10^6^ spores/ml in aseptic distilled water.

### Cultivation substrate

Vermiculite was used as the germination substrate of *S. miltiorrhiza* seeds. After 30 days of germination, *S. miltiorrhiza* seedlings were transplanted to 1:1 (v/v) mixture of paddy soil and vermiculite. The paddy soil contained organic matter (0.49 g·kg^-1^), total N (3.85 g·kg^-1^), total P (8.43 g·kg^-1^), available P (2.27 mg·kg^-1^), total K (28.43 g·kg^-1^), available K (8.71 mg·kg^-1^), available Zn (0.07 mg·kg^-1^), available Mn (0.74 mg·kg^-1^), available Fe (1.6 mg·kg^-1^), and available Cu (0.13 mg·kg^-1^), with a pH value of 8.7. The substrate was sterilized at 121°C for 2 hours before use.

### Experimental design

*S. miltiorrhiza* seeds were surface-sterilized in 75% ethanol for 1 min, soaked in 2% (V/V) NaClO for 10 min, and then rinsed with sterile water for 5 min. Germination substrate was autoclaved vermiculite. *S. miltiorrhiza* in AM treatment were pre-inoculated with *G.* versiforme, i.e., 100 g (equivalent to ~1250 spores) of AMF inoculum was mixed with 1 kg vermiculite. In NM treatment, an equal amount of autoclaved AMF inoculum was mixed with the vermiculite.

Thirty days after sowing, the mycorrhizal colonization of *S. miltiorrhiza* was assessed. *S. miltiorrhiza* seedlings were transplanted into square pots (7 cm × 7 cm), and inoculated with *F. oxysporum*. Four treatments were designed (NM-Fo, NM+Fo, AM-Fo, and AM+Fo): (1) NM-Fo: non-mycorrhizal *S. miltiorrhiza* inoculated with heat-killed pathogen; (2) NM+Fo: non-mycorrhizal *S. miltiorrhiza* inoculated with pathogen; (3) AM-Fo: mycorrhizal *S. miltiorrhiza* inoculated with heat-killed pathogen; (4) AM+Fo: mycorrhizal *S. miltiorrhiza* inoculated with pathogen. Each treatment included 60 pots. Seedlings were incubated in 5 mL spore suspension for 30min. Control *S. miltiorrhiza* were treated with 5 mL sterilized spore suspension for 30 min. Experiments were conducted in a greenhouse (30°C, 14L:10D photoperiod), with a photon flux density of 350 photon µmol·m^−2^·s^−1^ (photosynthetic active radiation).

### Assessment of AMF colonization

AMF colonization was measured 30 days after germination. The roots of mycorrhizal *S. miltiorrhiza* were cut into 1 cm long sections and then stained with Trypan Blue following the protocol published previously (Phillips and Hayman, 1970). AMF colonization of *S. miltiorrhiza* was determined as described previously (Giovannetti and Mosse, 1980).

### Disease incidence measured

Seven days after pathogen inoculation, disease incidence and disease index were measured. Disease incidence was calculated as the percentage of diseased *S. miltiorrhiza*. Disease severity was estimated using a Disease Index (DI) calculated as disease grades 0–5: 0, no symptoms; 1, growth delayed and no significant necrosis or atrophy of shoots and roots; 2, light chlorosis and necrosis on shoots and roots; 3, medium chlorosis and necrosis on shoots and roots; 4, high chlorosis and necrosis on shoots and roots; and 5, failed seedlings (Soudani et al., 2022). Disease incidence, disease index, and control efficacy were calculated using the following formulas:

$Disease\;Incidence=\frac{diseased\;plants}{sum\;of\;plants}\times 100\%$

$Disease\;Index=\frac{\sum^{}\left(disease\;grade\times number\;of\;diseased\;plants\right)}{maximum\;disease\;grades\times number\;of\;plants\;sample}\times 100\%$


$$Control\;Efficacy=\frac{DI\;\left(NM+Fo\right)-DI\;\left(AM+Fo\right)}{DI\;\left(NM+Fo\right)}\times 100\%$$

### Assessment of plant growth

Thirty days after pathogen inoculation, *S. miltiorrhiza* seedings were removed from the soil, the shoots and roots were separated, and the fresh weights of both the shoots and roots were recorded.

### Root system measurement

Thirty days after pathogen inoculation, the roots of *S. miltiorrhiza* were scanned with an Epson Expression/STD 4800 scanner (Seiko Epson Corporation, Nagano, Japan), and the root length, root projArea, and root surfArea were derived with WinRHIZO image analysis software (Regent Instruments Inc., Quebec, QC, Canada).

### Chlorophyll fluorescence measurement

The chlorophyll fluorescence parameters were determined 30 days after pathogen inoculation. A dual-PAM-100 device (Heinz Walz, Effeltrich, Germany) was used to measure the Chlorophyll fluorescence parameters of the two uppermost leaves of *S. miltiorrhiza* at 25°C according the previous published protocols (Ritchie and Bunthawin, 2010). Before measurement, the minimal fluorescence in the dark-adapted state (*F_0_*) was recorded after the plants were kept in the darkness for 30 min. The maximal fluorescence in the dark-adapted state (*F_m_*), the maximal fluorescence (*F_m_’*), the minimal fluorescence in the light-adapted state (*F_0_’*), and the steady-state fluorescence (*F_s_*) of leaves were determined following the previously described methods (Gong et al., 2013). The chlorophyll fluorescence parameters Φ_PSII_, *F_v_*/*F_m_*, q_P_, and q_N_ were as described (Zai et al., 2012; Sowik et al., 2016).

### Chlorophyll measurement

Thirty days after pathogen inoculation, chlorophyll content was measured as described previously (Gregor and Maršálek, 2004). Approximately 0.05 g fresh leaves of *S. miltiorrhiza* were ground into fine powder and 8 mL 95% ethanol was added. Samples were stored in the dark for 48 h. The absorption of the continuation filtrate was measured at 665 nm, 649 nm, and 470 nm and the content of chlorophyll was calculated according to the following formulas:

C_a_ = 13.95A665 - 6.88A649, C_b_ = 24.96A649 - 7.32A665, C_Chl_ = Ca + Cb, C_Car_ = (1000A470 - 2.05Ca - 114.8Cb)/245

Chlorophyll a content = C_a_ × V/W, Chlorophyll b content=C_b_ × V/W, Total Chlorophyll content = C_Chl_ × V/W, Carotenoid content = C_Car_ × V/W

### Content of soluble protein measurement

Thirty days after pathogen inoculation, soluble protein content was determined according to the previously published method (Yen and Pratap-Singh, 2021). A standard curve was constructed using different concentrations (0-2 mg·mL^-1^) of bovine serum albumin (BSA) to estimate of protein content.

### Activities of defense-related enzymes

The activities of defense-related enzymes were detected five days following infection. Approximately 0.1 g root samples of *S. miltiorrhiza* were ground into fine powder in liquid nitrogen and were extracted with 2 mL 0.05 M sodium acetate buffer (pH 5.0). Extracts were centrifuged at 12,000 *g* for 15 min at 4°C and the supernatant fractions were used to assay enzyme activity. PAL activity was analyzed as Mozzetti et al. (1995) described. β-1,3-Glucanase activity was assayed by the laminarin-dinitro salicylic acid method (Pan, 1991). Chitinase activity was analyzed as Boller and Mauch (1988) described.

### Expression of defense-related genes

The expression levels of defense-related genes, *SmLOX* (JX297420.1), *SmAOS*, *SmAOC* (HM156740.1), *SmOPR* (MN125491.1), *SmJAR*, *SmPDF2.1* (OP066222), *SmPAL* (DQ408636.1), *SmNPR1*, *SmPR1*, and *SmPR10* (KF877034.1), were measured by qRT-PCR three days after pathogen inoculation. To do this, 0.1 g root samples were ground into fine powder in liquid nitrogen and total RNA was extracted using the RNeasy Plus Mini kit (Qiagen, Germany). Reverse transcription was performed using PrimeScript™ Reverse Transcriptase (TaKaRa, Japan). Primer Premier 5 software used to design the primers as shown in **Table S1** and qRT-PCR analysis was conducted using SYBR^®^ Premix Ex Taq™ II (TaKaRa, Japan), with *SmActin* (DQ243702) as a reference gene using a LightCycler 480 real-time PCR system (Roche, Switzerland). C_T_ values were calculated to analyze the relative expression levels using the 2^-ΔΔCt^ method (Guo et al., 2016).

### Statistical analysis

All data were analyzed using IBM SPSS Statistics 24. Results are presented as the mean values ± standard deviation (SD). Data were analyzed with two-way ANOVA followed by Tukey’s test and differences were reported as significant for values of *P* < 0.05.

## Results

### Induction of disease resistance by Mycorrhizal colonization

**Figure 1:**
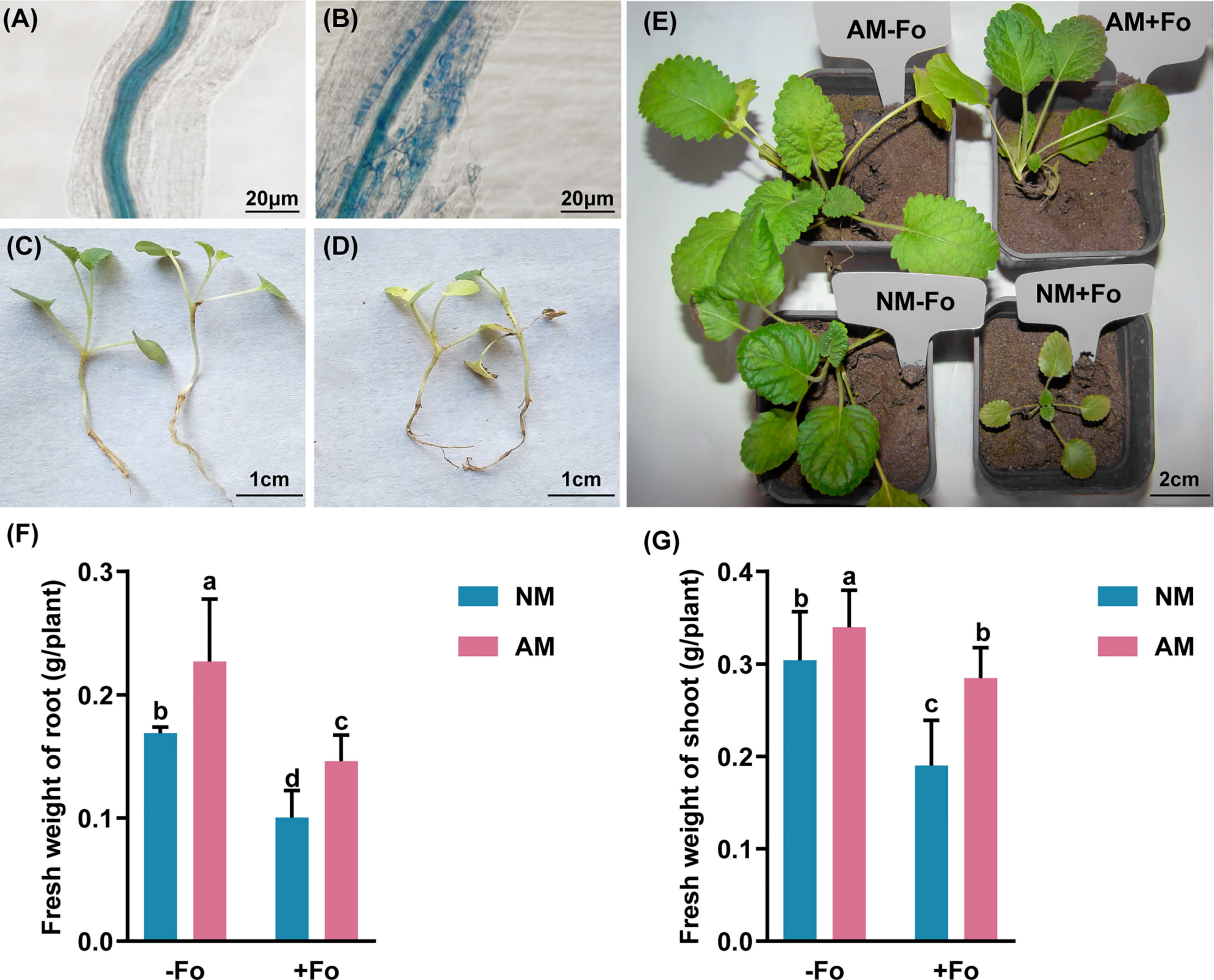
*G versiforme* alleviates disease of *S. miltiorrhiza* infected with *F oxysporum*. **(A, B)** Uncolonized roots **(A)** and colonized roots **(B)** by *G versiforme*. The photos were taken 30 days after mycorrhizal inoculation. **(C, D)** *S. miltiorrhiza* seedlings without pathogen inoculation **(C)** and diseased *S. miltiorrhiza* seedlings infected with the pathogen **(D)**. The photos were taken 7 days after inoculating with *F oxysporum*. **(E)** *S. miltiorrhiza* plants of four treatments 30 days after inoculating with *F oxysporum*. **(F, G)** Fresh weight of shoot **(F)** and root **(G)** of *S. miltiorrhiza* 30 days after pathogen inoculation. Four treatments included: (1) NM-Fo: non-mycorrhizal *S. miltiorrhiza* inoculated with heat-killed pathogen inoculation; (2) NM+Fo: non-mycorrhizal *S. miltiorrhiza* inoculated with pathogen; (3) AM-Fo: mycorrhizal *S. miltiorrhiza* inoculated with heat-killed pathogen; (4) AM+Fo: mycorrhizal *S. miltiorrhiza* inoculated with pathogen. Values are means ± SD from four sets of independent experiments with 30 pots per treatment for each set of experiments. Different lowercase letters indicate significant differences between different treatments according to two-way ANOVA followed by Tukey’s test for multiple comparisons (*P* < 0.05).

Mycorrhizal colonization was examined 30 days post-inoculation. Among the *S. miltiorrhiza* treated with *G. versiforme* (AM treatment), 83.33 ± 3% were successfully colonized by *G. versiforme* (**Figures 1A, B**, **Table 1**). There was no fungal structure in the roots of plants in the NM treatment. The results showed that *S. miltiorrhiza* was successfully colonized by the AMF and the pathogen could be inoculated later.

**Table 1:** Mycorrhizal colonization, disease incidences, and indices of *S. miltiorrhiza* inoculated with *F. oxysporum*.

Treatment	Mycorrhizal colonization (%)	Disease incidence (%)	Disease index (%)	Control efficacy (%)
NM-Fo	0	0c	0c	0
NM+Fo		48.3 ± 7.6a	41.5 ± 3.3a	0
AM-Fo	83.33 ± 3	0c	0c	0
AM+Fo		18.3 ± 2.8b	15.5 ± 2.3b	62.6

Values are means ± SD from four sets of independent experiments with 60 pots per treatment for each set of experiments. Different lowercase letters indicate significant differences between different treatments according to two-way ANOVA followed by Tukey’s test for multiple comparisons (P < 0.05).

No disease symptoms were found in the two groups without inoculation of the pathogen (**Figures 1C, E**). Disease symptoms of *S. miltiorrhiza* infected with *F. oxysporum* exhibited dwarfish stem, yellow and smallish leaves, and generally withered plants (**Figures 1D, E**). Pre-inoculation of *S. miltiorrhiza* with the *G. versiforme* significantly decreased the disease incidence and disease severity of Fusarium wilt compared to the plants in the NM+Fo treatment. The disease incidence and disease index of the NM+Fo treatment were 48.3% and 41.5%, while those of the AM+Fo treatment were only 18.3% and 15.5% after seven days of pathogen inoculation (**Table 1**). Disease incidence was reduced by 62.1% in mycorrhizal plants. Mycorrhizal plants had significantly decreased disease symptoms compared to non-mycorrhizal inoculated plants 45 days after pathogen infection (**Figure 1E**). The control efficacy of AMF pre-inoculation was 62.6% (**Table 1**).

### *G. versiforme* alleviated the retarded growth of *S. miltiorrhiza* resulting from *F. oxysporum* infection

*G. versiforme* colonization significantly increased the fresh weight of shoots and roots by 11.74% and 34.56%, respectively (**Figures 1F, G**). In contrast, *F. oxysporum* decreased the shoot biomass and root biomass by 37.5% and 40.6%, respectively (**Figures 1F, G**). Mycorrhizal plants promoted the accumulation of plant biomass relative to non-mycorrhizal plants after inoculation with the pathogen (**Figure 1E**). Compared to NM+Fo treatment, pre-inoculation with AMF (AM+Fo treatment) increased the fresh weight of shoots and roots by 49.8% and 45.7%, respectively.

### *G. versiforme* improved root morphology of *S. miltiorrhiza* infected with *F. oxysporum*

**Figure 2:**
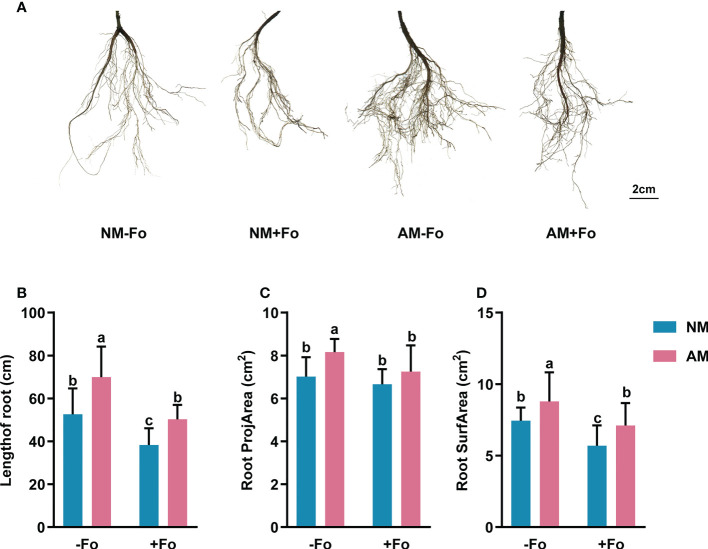
Root morphology of *S. miltiorrhiza* in the four treatments. **(A)** Root scans of *S. miltiorrhiza* in AM+Fo treatment and NM+Fo treatment. Length of root **(B)**, root projArea **(C)**, and root surfArea **(D)** of *S. miltiorrhiza* 30 days after pathogen inoculation. Four treatments included: (1) NM-Fo: non-mycorrhizal *S. miltiorrhiza* inoculated with heat-killed pathogen inoculation; (2) NM+Fo: non-mycorrhizal *S. miltiorrhiza* inoculated with pathogen; (3) AM-Fo: mycorrhizal *S. miltiorrhiza* inoculated with heat-killed pathogen; (4) AM+Fo: mycorrhizal *S. miltiorrhiza* inoculated with pathogen. Values are means ± SD from four sets of independent experiments with 30 pots per treatment for each set of experiments. Different lowercase letters indicate significant differences between different treatments according to two-way ANOVA followed by Tukey’s test for multiple comparisons (*P* < 0.05).

The results of root scanning showed that the *F. oxysporum* infection seriously damaged the root system of *S miltiorrhiza*, resulting in less fibrous roots and root vascular blocking, while mycorrhizal colonization greatly promoted the development of root system (**Figure 2A**). Mycorrhizal *S. miltiorrhiza* partially resisted root damage caused by pathogen infection (**Figure 2A**). *G. versiforme* colonization significantly increased the length of root by 32.80%, root projArea by 16.27%, and root surfArea by 18.18%, but pathogen infection decreased those of *S. miltiorrhiza* (**Figures 2B–D**). Pre-inoculating AMF decreased the loss of root biomass caused by pathogen infection. The length of root and root surfArea of *S. miltiorrhiza* in NM+Fo treatment were significantly lower than those of *S. miltiorrhiza* in AM+Fo treatment (**Figures 2B, D**).

### *G. versiforme* improved photosynthesis of *S. miltiorrhiza* infected with *F. oxysporum*

*F. oxysporum* infection decreased the photosynthesis-related parameters Φ_PSII_ and *F_v_*/*F_m_* of non-mycorrhizal *S. miltiorrhiza* by 20.2% and 13%, respectively. While 10% decreased on Φ_PSII_ and no significant difference in *F_v_*/*F_m_* (**Table 2**) of mycorrhizal *S. miltiorrhiza*. Pathogen inoculation also decreased the q_P_ and q_N_, but there was no significant difference between the four treatments (**Table 2**).

**Table 2:** Chlorophyll fluorescence parameters in leaves of *S. miltiorrhiza* seedlings.

Treatment	Φ_PSII_	*F_v_*/*F_m_*	q_P_	q_N_
NM-Fo	0.450 ± 0.028a	0.6312 ± 0.074a	0.860 ± 0.147a	0.556 ± 0.147a
NM+Fo	0.359 ± 0.022b	0.548 ± 0.073b	0.794 ± 0.065a	0.523 ± 0.065a
AM-Fo	0.439 ± 0.019a	0.650 ± 0.038a	0.854 ± 0.067a	0.555 ± 0.067a
AM+Fo	0.395 ± 0.014b	0.627 ± 0.031a	0.798 ± 0.091a	0.536 ± 0.091a

Values are means ± SD from four sets of independent experiments with 15 plants. Different lowercase letters indicate significant differences between different treatments according to two-way ANOVA followed by Tukey’s test for multiple comparisons (P < 0.05).

The content of Chlorophyll a, Chlorophyll b, and total Chlorophyll of *S. miltiorrhiza* in the NM+Fo treatment were significantly decreased by 10%, 11%, and 15% compared with NM-Fo treatment. However, the above parameters were not decreased by pathogen inoculation in mycorrhizal *S. miltiorrhiza* (**Table 3**). In addition, AMF colonization significantly increased the content of carotenoid (**Table 3**).

**Table 3:** Content of Chlorophyll a, Chlorophyll b, Carotenoid, and total Chlorophyll in leaves of *S. miltiorrhiza* seedlings.

Treatment	Chlorophyll a (mg/g)	Chlorophyll b (mg/g)	Carotenoid (mg/g)	Total Chlorophyll (mg/g)
NM-Fo	0.846 ± 0.019a	0.303 ± 0.008a	0.335 ± 0.012bc	1.318 ± 0.012a
NM+Fo	0.759 ± 0.011b	0.269 ± 0.007b	0.315 ± 0.005c	1.117 ± 0.017b
AM-Fo	0.813 ± 0.018ab	0.304 ± 0.009a	0.363 ± 0.008a	1.281 ± 0.023a
AM+Fo	0.790 ± 0.022ab	0.285 ± 0.018ab	0.344 ± 0.039ab	1.233 ± 0.038ab

Values are means ± SD from four sets of independent experiments with 15 plants. Different lowercase letters indicate significant differences between different treatments according to two-way ANOVA followed by Tukey’s test for multiple comparisons (P < 0.05).

### *G. versiforme* improved the protein content of *S. miltiorrhiza* infected with *F. oxysporum*

**Figure 3:**
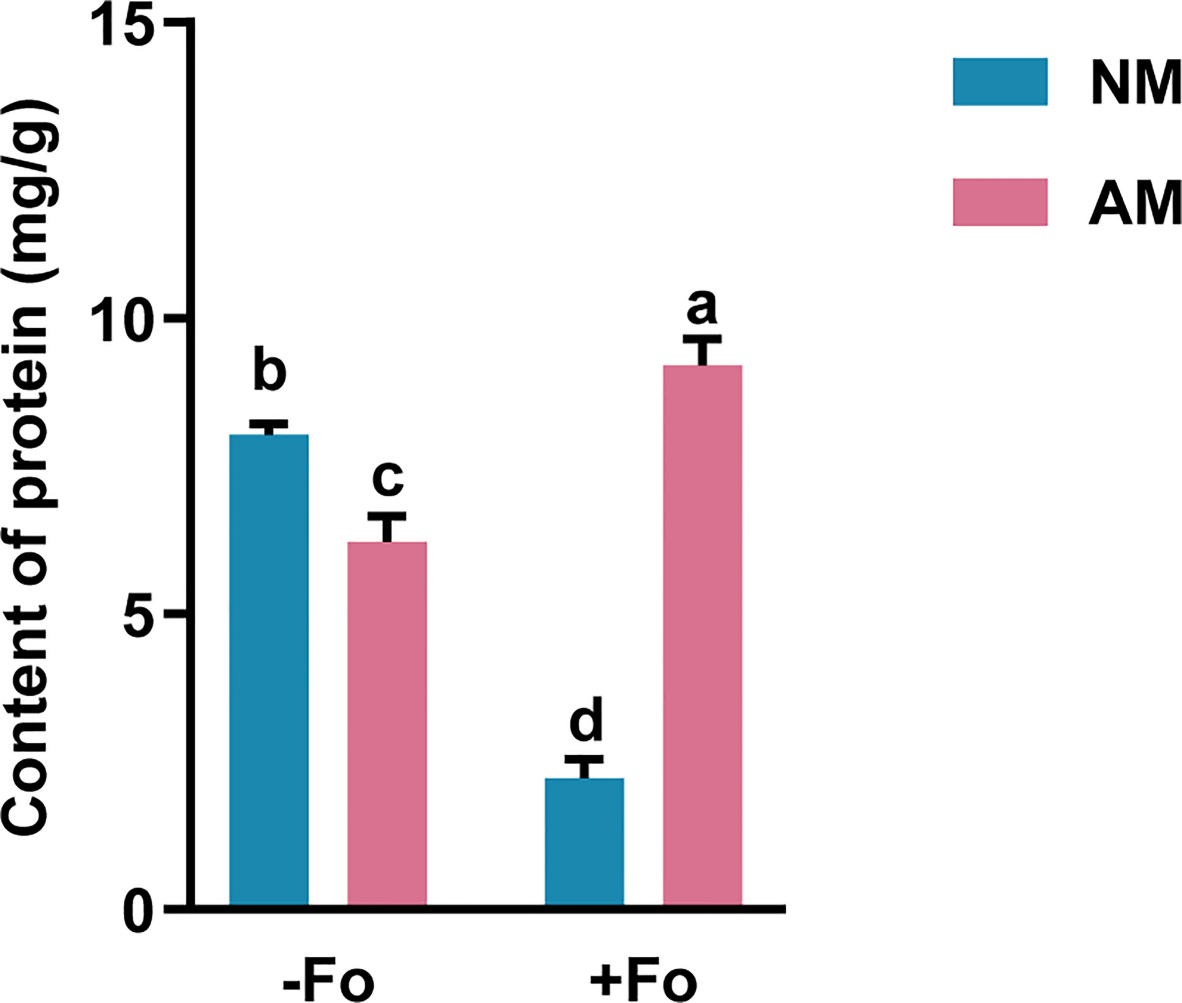
Protein content in the roots of *S. miltiorrhiza* from the four treatments. The protein content was measured 30 days after pathogen inoculation. Data are mean values ± SD; significant differences (*P* < 0.05 using Tukey’s test) among treatments in the same column are indicated by different letters. Four treatments included: (1) NM-Fo: non-mycorrhizal *S. miltiorrhiza* inoculated with heat-killed pathogen inoculation; (2) NM+Fo: non-mycorrhizal *S. miltiorrhiza* inoculated with pathogen; (3) AM-Fo: mycorrhizal *S. miltiorrhiza* inoculated with heat-killed pathogen; (4) AM+Fo: mycorrhizal *S. miltiorrhiza* inoculated with pathogen. Values are means ± SD from four sets of independent experiments with 15 plants per treatment. Different lowercase letters indicate significant differences between different treatments according to two-way ANOVA followed by Tukey’s test for multiple comparisons (*P* < 0.05).

Mycorrhizal colonization significantly reduced the content of soluble protein in the roots of *S. miltiorrhiza* by 22.6% compared with non-mycorrhizal plants (**Figure 3**). *F. oxysporum* infection significantly reduced the protein content in non-mycorrhizal *S. miltiorrhiza* by 72.4% but increased protein content by 48% in mycorrhizal *S. miltiorrhiza* (**Figure 3**).

### Induction of defense-related enzymes in Mycorrhizal *S. miltiorrhiza* by pathogen infection

To determine the effects of AMF colonization on defense responses in *S. miltiorrhiza*, the levels of three defense-related enzymes, PAL, β-1,3-glucanase, and chitinase, were analyzed in the roots of *S. miltiorrhiza* after pathogen infection.

**Figure 4:**
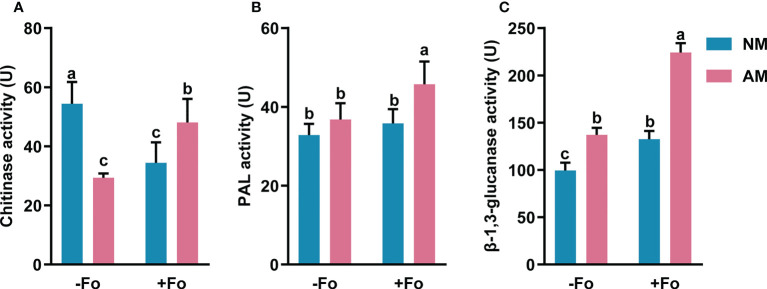
The activity of chitinase **(A)**, PAL **(B)**, and β-1,3-glucanase **(C)** in the roots of *S. miltiorrhiza* five days after pathogen inoculation. Four treatments included: (1) NM-Fo: non-mycorrhizal *S. miltiorrhiza* inoculated with heat-killed pathogen inoculation; (2) NM+Fo: non-mycorrhizal *S. miltiorrhiza* inoculated with pathogen; (3) AM-Fo: mycorrhizal *S. miltiorrhiza* inoculated with heat-killed pathogen; (4) AM+Fo: mycorrhizal *S. miltiorrhiza* inoculated with pathogen. Values are means ± SD from four sets of independent experiments with 15 plants per treatment. Different lowercase letters indicate significant differences between different treatments according to two-way ANOVA followed by Tukey’s test for multiple comparisons (*P* < 0.05).

The PAL activity of mycorrhizal and pathogen-infected *S. miltiorrhiza* (AM+Fo treatment) was significantly increased by 39% compared with that of control NM treatment *S. miltiorrhiza* (**Figure 4A**). However, inoculation of *S. miltiorrhiza* with AMF or pathogen alone did not significantly enhance PAL activity in the roots of *S. miltiorrhiza* (**Figure 4A**).

Inoculation of AMF or pathogen alone significantly increased β-1,3-glucanase activity by 28% and 34%, respectively, while inoculation of *S. miltiorrhiza* with both AMF and pathogen increased β-1,3-glucanase activity by 125% (**Figure 4B**).

Unlike the increased activities of PAL and β-1,3-glucanase, chitinase activity significantly decreased by 39% and 45% after AMF colonization or pathogen infection, respectively. However, there was a smaller drop (11.55%) in the activity of chitinase after AMF and pathogen dual inoculation (**Figure 4C**).

Overall, mycorrhizal *S. miltiorrhiza* treatment showed higher increases in three enzymes activities after pathogen infection, especially PAL and β-1,3-glucanase, suggesting that mycorrhizal pre-inoculation enhanced the activities of these enzymes in the roots of *S. miltiorrhiza* upon pathogen infection.

### Mycorrhizal colonization induced transcription of defense-related genes

**Figure 5:**
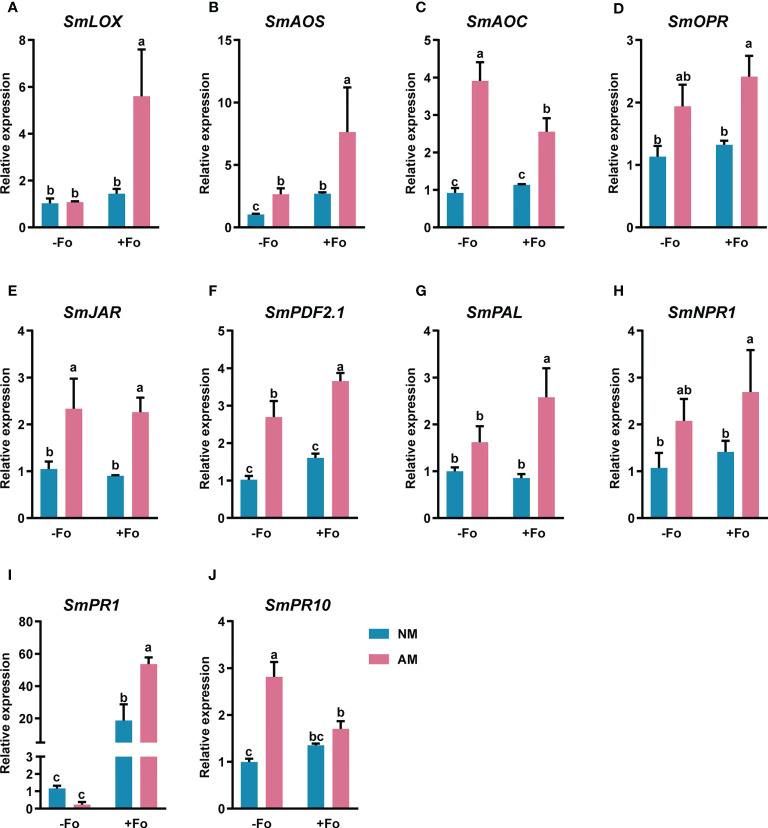
Relative expression levels of *SmLOX* **(A)**, *SmAOS* **(B)**, *SmAOC* **(C)**, *SmOPR* **(D)**, *SmJAR* **(E)**, *SmPDF2.1* **(F)**, *SmPAL* **(G)**, *SmNPR1* **(H)**, *SmPR1* **(I)**, and *SmPR10* **(J)** in the roots of S. miltiorrhiza three days after pathogen inoculation. Four treatments included: (1) NM-Fo: non-mycorrhizal *S. miltiorrhiza* inoculated with heat-killed pathogen inoculation; (2) NM+Fo: non-mycorrhizal *S. miltiorrhiza* inoculated with pathogen; (3) AM-Fo: mycorrhizal *S. miltiorrhiza* inoculated with heat-killed pathogen; (4) AM+Fo: mycorrhizal *S. miltiorrhiza* inoculated with pathogen. Values are means ± SD from four sets of independent experiments with 15 plants per treatment. Different lowercase letters indicate significant differences between different treatments according to two-way ANOVA followed by Tukey’s test for multiple comparisons (*P* < 0.05).

To determine whether the transcript induction of defense-related genes was enhanced by mycorrhizal colonization, gene expression was analyzed from *S. miltiorrhiza* roots three days after pathogen inoculation using real-time RT-PCR. The amplification efficiency of the primer pairs ranged from 90 to 110% (**Table S1**, **Figure S2**). These primers were used for quantitive analysis of the transcriptional activity of defense-related genes. The JA synthesis pathway genes, *SmLOX*, *SmAOS*, *SmAOC*, and *SmOPR*, were significantly up-regulated by 443%, 653%, 178%, and 113%, respectively, in mycorrhizal *S. miltiorrhiza* roots after pathogen infection (**Figures 5A–D**). However, pathogen infection alone did not induce these gene transcription. Similarly, the JA signaling pathway gene, *SmJAR*, and the markers of the JA defense-response pathway, *SmPDF2.1*, were upregulated by 116% and 257%, respectively, in mycorrhizal *S. miltiorrhiza* roots after pathogen infection (**Figures 5E, F**). In addition, inoculation AMF alone up-regulated the transcripts of *SmAOS*, *SmAOC*, *SmJAR*, and *SmPDF2.1*. by 156%, 325%, 123%, and 163%, respectively (**Figures 5B, C, E, F**).

*SmPAL*, the key gene involved in the biosynthesis of SA (Shine et al., 2016), and *SmNPR1*, a master regulator of SA (Tada et al., 2008), were significantly up-regulated by 156% and 151%, respectively, in mycorrhizal *S. miltiorrhiza* roots after pathogen infection (**Figures 5G, H**). However, there were no expression changes in response to either mycorrhizal colonization or pathogen infection alone. *SmPR1* and *SmPR10*, encode pathogenesis-related proteins and were significantly up-regulated in mycorrhizal *S. miltiorrhiza* roots after pathogen infection (**Figures 5I, J**). After pathogen infection, *SmPR1* was 45-fold up-regulated in mycorrhizal *S. miltiorrhiza* roots, while 15-fold up-regulated in non-mycorrhizal *S. miltiorrhiza* roots (**Figure 5I**).

## Discussion

Fusarium wilt has become a major disease of *S. miltiorrhiza* and is a major limiting factor for cultivation. We showed that the Fusarium wilt caused by *F. oxysporum* can be alleviated through mycorrhizal pre-inoculation. Pre-inoculation of *S. miltiorrhiza* with *G. versiforme* significantly decreased disease incidence (from 48.3% to 18.3%) and disease index (from 41.5% to15.5%) of Fusarium wilt compared to *S. miltiorrhiza* without mycorrhizal colonization (**Table 1**). *F. oxysporum* infection reduced the shoot and root biomass of non-mycorrhizal *S. miltiorrhiza* by 37.5% and 40.6%, however, *G. versiforme* pre-inoculation reduced the loss of shoot biomass to 16.19% and root biomass to 35.68% (**Figures 1F, G**). The results were in accordance with previous reports that AMF colonization alleviates alfalfa leaf spots caused by *Phoma medicaginis* (Li et al., 2021), and that *Funneliformis mosseae* significantly alleviates early blight disease in tomato caused by *Alternaria solani* Sorauer (Song et al., 2015).

Roots allow plants to absorb nutrients and water. Infection of *S. miltiorrhiza* by *F. oxysporum* leads to Fusarium wilt, with symptoms including less fibrous roots and root vascular blocking, decreasing absorption of nutrients and water and resulting in plant wilting and death (Yang et al., 2013; Chen et al., 2017a). We found that *G. versiforme* increased the length of root by 32.80%, root projArea by 16.27%, and root surfArea by 18.18% of *S. miltiorrhiza* (**Figures 2A–D**). Root structure is key to determining a plant’s ability to effectively explore soils (Dorlodot et al., 2007). AMF improves plant nutrition, and this could contribute to increased plant tolerance and compensation for root damage caused by the pathogen (Cordier et al., 1998). The increased nutrition and fitness of mycorrhizal plants likely serve as systemic protection mechanisms against pathogen attack (Fritz et al., 2006). Many studies have shown that the major benefit of AMF colonization is its effect on the host root system (Gutjahr et al., 2009; Vos et al., 2013). Consistently, the improved root system structure we observed here appears to be a key factor in the disease resistance of *S. miltiorrhiza* induced by *G. versiforme*.

Photosynthesis not only provides nutrients for plant growth, but also produces defense-related substances to counter pathogens (Serrano et al., 2016). Suppressing photosynthesis is also a strategy for successful infection of pathogens. *F_v_*/*F_m_* and Φ_PSII_ are important indicators of the photosynthetic apparatus and are widely used to assess plant-pathogen interactions (Wang et al., 2018). Consistent with our findings (**Table 2**), a previous study found that pathogen infection significantly reduced *F_v_*/*F_m_* and Φ_PSII_ in plants not inoculated with AMF, but had no effect in mycorrhizal plants (Wang et al., 2018). Here, *F. oxysporum* infection decreased the photosynthesis-related parameters *F_v_*/*F_m_* and Φ_PSII_ by 20.2% and 13% in non-mycorrhizal *S. miltiorrhiza*, respectively, while Φ_PSII_ only decreased by 10% and *F_v_*/*F_m_* did not decrease in mycorrhizal *S. miltiorrhiza* (**Table 2**). In plants, carotenoids play vital roles in photosynthesis as light-harvesting pigments and photo-protective compounds (Gupta and Hirschberg, 2022). Previous study demonstrated a positive correlation between carotenoids and photosynthetic rate (Lobato et al., 2010). In this study, the pathogen infection significantly increased the carotenoid content in mycorrhizal *S. miltiorrhiza*. Together, these results suggest that the disease resistance of *S. miltiorrhiza* can be improved by improving photosynthesis.

The activity of defense-related enzymes (e.g., PAL, chitinase, and β-1,3-glucanase) can be enhanced when systemic resistance is activated (Hura et al., 2014; Jain and Choudhary, 2014; Eke et al., 2016; Gharbi et al., 2017). Here, we found that, after pathogen infection, the activities of PAL, chitinase, and β-1,3-glucanase showed greater increases in the roots of mycorrhizal *S. miltiorrhiza* than in non-mycorrhizal *S. miltiorrhiza* plants (**Figures 4A–C**). These enzymes are crucial components in plant resistance to biotic diseases (Funnell et al., 2004). PAL is the key enzyme in the biosynthesis of multiple antimicrobial compounds (phenolic acid, flavonoids), lignin (a rapidly deposited physical barrier), and salicylic acid, three compunds that are related to plant resistance (Wang et al., 2019). Chitinase and β-1,3-glucanase can degrade pathogenic fungal cellular components to inactivate fungi, and also produce monomers to further stimulate plant defense responses (Anguelova-Merhar et al., 2001; Doxey et al., 2007; Kumar et al., 2018). Our results showed that AMF can trigger the expression of defense enzymes in the host plant, which was similar to the response of *F. oxysporum* infection (**Figures 4A–C**). Inoculating AMF or pathogen alone significantly increased β-1,3-glucanase activity, inhibited chitinase activity, and did not affect PAL activity in *S. miltiorrhiza* (**Figures 4A–C**). These results indicate that inoculation of AMF or infection with pathogen alone can stimulate β-1,3-glucanase-related defense responses, but do not affect chitin- and PAL-related defense responses. This result is in agreement with the report that mycorrhizal fungi initially trigger plant defense mechanisms similarly to a biotrophic pathogen (Paszkowski, 2006). Song et al. (2015) found that AMF inoculation itself did not affect most enzyme activities, but after pathogen attack AMF pre-inoculation induces tomato plants to produce a defense response of four defense-related enzymes. Our results showed that upon pathogen attack (AM+Fo treatment), AMF pre-inoculation strongly induced the activities of PAL and β-1,3-glucanase by 39.23% and 125.18%, respectively (**Figures 4A, B**). PAL and β-1,3-glucanase activities in the AM+Fo treatment were the highest among all treatments. Pre-inoculation of AMF can alleviate the inhibitory effect on chitinase activity caused by the pathogen. Overall, pre-inoculation with *G. versiforme* inhibits pathogen infection by increasing the activities of PAL, β-1,3-glucanase, and chitinase.

Disease resistance in plants is tightly regulated through an interlinked network of JA and SA signaling pathways (Song et al., 2015). The JA signaling pathway plays an important role in plant defense response, and *SmLOX*, *SmAOS*, *SmAOC*, and *SmOPR* are important genes in JA biosynthes. Plant defensins (PDFs) are a family of small cysteine-rich basic proteins (García-Olmedo et al., 1998). *SmPDF2.1*, a gene encoding plant defensin, is a marker of the jasmonate (JA) defense-response pathway (Hanks et al., 2005). The stronger induction of these genes in mycorrhizal plants after pathogen infection suggested that mycorrhizal colonization activates the JA signaling pathway and enhances the resistance of *S. miltiorrhiza* to *F. oxysporum*. This is consistent with previous studies that mycorrhizal colonization enhances resistance to early blight in tomato by initiating a systemic defense response and that the JA signaling pathway is critical in the mycorrhizal-initiated disease resistance process (Song et al., 2015). *SmPAL* is a key gene involved in the biosynthesis of SA (Shine et al., 2016), and *SmNPR1* is a master regulator of SA (Tada et al., 2008). The induction of *SmPR1* and *SmPR10* indicates that mycorrhizal colonization provokes SA signaling pathways upon pathogen attack. *PR* genes are usually used as marker genes of the acquisition of systemic resistance in plants (Mitsuhara et al., 2008) and the levels of PR proteins are used as an indicator of defense responses (Song et al., 2015). Pathogenesis-related 1 (PR1) protein is a commonly used reporter of SA-activated defense responses in plants (Pečenková et al., 2022). Consistent with our studies, many studies reported that mycorrhizal colonization induced the transcription of *PR* genes (Ismail and Hijri, 2012; Li et al., 2021).

## Conclusion

Pre-inoculation of *S. miltiorrhiza* with the AMF, *G. versiforme*, enhanced resistance to Fusarium wilt by priming the systemic defense response. Mycorrhizal colonization improved the root structure and photosynthesis capacity of *S. miltiorrhiza* to reduce disease incidence. Infection with the pathogen alone could evade the PAL- and chitinase-related defense responses, however pre-inoculation of *S. miltiorrhiza* with AMF strongly induced PAL-, β-1,3-glucanase-, and chitinase-related defense responses upon pathogen attack. JA and SA signaling pathways are key components of the plant defense response, and were strongly activated by pre-inoculation of AMF upon pathogen attack.

## Data availability statement

The original contributions presented in the study are included in the article/**Supplementary Material**. Further inquiries can be directed to the corresponding author.

## Author contributions

MC and GY designed the experiments, which were performed by CP, HZ, and SL. CP wrote the manuscript and analyzed the results. YG, ZC, WG, and YS revised the manuscript. MC and LH provided the funding. All authors contributed to the article and approved the submitted version.

## Funding

The Scientific and Technological Innovation Project of China Academy of Chinese Medical Sciences (CI2021A03906) and the National Natural Science Foundation of China (81773849, 82173931, 81803658) supported this work.

## Conflict of interest

The authors declare that the research was conducted in the absence of any commercial or financial relationships that could be construed as potential conflicts of interest.

## Publisher’s note

All claims expressed in this article are solely those of the authors and do not necessarily represent those of their affiliated organizations, or those of the publisher, the editors and the reviewers. Any product that may be evaluated in this article, or claim that may be made by its manufacturer, is not guaranteed or endorsed by the publisher.

## References

[B1] Ajit V. Ram P. Narendra T. (2017). Mycorrhiza-Nutrient Uptake, Biocontrol, Ecorestoration. (Berlin: Springer Cham). C1–C1. doi: 10.1007/978-3-319-68867-1_27

[B2] Anguelova-Merhar V. S. Westhuizen A. J. Pretorius Z. A. (2001). β-1,3-Glucanase and chitinase activities and the resistance response of wheat to leaf rust. J. Phytopathol. 149, 381–384. doi: 10.1111/j.1439-0434.2001.tb03866.x

[B3] Ankati S. Srinivas V. Pratyusha S. Gopalakrishnan S. (2021). *Streptomyces consortia*-mediated plant defense against fusarium wilt and plant growth-promotion in chickpea. Microb. Pathogenesis 157, 104961. doi: 10.1016/j.micpath.2021.104961 34033892

[B4] Bai Y. Kissoudis C. Yan Z. Visser R. G. F. van der Linden G. (2018). Plant behaviour under combined stress: tomato responses to combined salinity and pathogen stress. Plant J. 93, 781–793. doi: 10.1111/tpj.13800 29237240

[B5] Bellincampi D. Cervone F. Lionetti V. (2014). Plant cell wall dynamics and wall-related susceptibility in plant–pathogen interactions. Front. Plant Sci. 5. doi: 10.3389/fpls.2014.00228 PMC4036129 24904623

[B6] Boller T. Mauch F. (1988). Biomass part b: Lignin, pectin, and chitin. Methods Enzymol. 161, 430–435. doi: 10.1016/0076-6879(88)61052-4 3226292

[B7] Chen H. Wu H. Yan B. Zhao H. Liu F. Zhang H.. (2018). Core microbiome of medicinal plant *Salvia miltiorrhiza* seed: A rich reservoir of beneficial microbes for secondary metabolism? Int. J. Mol. Sci. 19, 672. doi: 10.3390/ijms19030672 29495531 PMC5877533

[B8] Chen M. Yang G. Liu D. Li M. Qiu H. Guo L.. (2017a). Inoculation with *Glomus mosseae* improves the growth and salvianolic acid b accumulation of continuously cropped *Salvia miltiorrhiza* . Appl. Sci. 7, 692. doi: 10.3390/app7070692

[B9] Chen M. Yang G. Sheng Y. Li P. Qiu H. Zhou X.. (2017b). *Glomus mosseae* inoculation improves the root system architecture, photosynthetic efficiency and flavonoids accumulation of liquorice under nutrient stress. Front. Plant Sci. 08. doi: 10.3389/fpls.2017.00931 PMC5461296 28638391

[B10] Cordier C. Pozo M. J. Barea J. M. Gianinazzi S. Gianinazzi-Pearson V. (1998). Cell defense responses associated with localized and systemic resistance to *Phytophthora parasitica* induced in tomato by an arbuscular mycorrhizal fungus. Mol. Plant-Microbe Interact. 11, 1017–1028. doi: 10.1094/mpmi.1998.11.10.1017

[B11] de Lamo,. F. J. Takken F. L. W. (2020). Biocontrol by *Fusarium oxysporum* using endophyte-mediated resistance. Front. Plant Sci. 11. doi: 10.3389/fpls.2020.00037 PMC7015898 32117376

[B12] Dey M. Ghosh S. (2022). Arbuscular mycorrhizae in plant immunity and crop pathogen control. Rhizosphere 22, 100524. doi: 10.1016/j.rhisph.2022.100524

[B13] Dong X. Wang M. Ling N. Shen Q. Guo S. (2016). Potential role of photosynthesis-related factors in banana metabolism and defense against *Fusarium oxysporum* f. sp. cubense. Environ. Exp. Bot. 129, 4–12. doi: 10.1016/j.envexpbot.2016.01.005

[B14] Dorlodot S. Forster B. Pagès L. Price A. Tuberosa R. Draye X. (2007). Root system architecture: opportunities and constraints for genetic improvement of crops. Trends Plant Sci. 12, 474–481. doi: 10.1016/j.tplants.2007.08.012 17822944

[B15] Doxey A. C. Yaish M. W. F. Moffatt B. A. Griffith M. McConkey B. J. (2007). Functional divergence in the arabidopsis β-1,3-Glucanase gene family inferred by phylogenetic reconstruction of expression states. Mol. Biol. Evol. 24, 1045–1055. doi: 10.1093/molbev/msm024 17272678

[B16] Eke P. Chatue G. C. Wakam L. N. Kouipou R. M. T. Fokou P. V. T. Boyom F. F. (2016). Mycorrhiza consortia suppress the fusarium root rot (*Fusarium solani* f. sp. phaseoli) in common bean (*Phaseolus vulgaris* l.). Biol. Control 103, 240–250. doi: 10.1016/j.biocontrol.2016.10.001

[B17] Fritz M. Jakobsen I. Lyngkjær M. F. Thordal-Christensen H. Pons-Kühnemann J. (2006). Arbuscular mycorrhiza reduces susceptibility of tomato to *Alternaria solani* . Mycorrhiza 16, 413–419. doi: 10.1007/s00572-006-0051-z 16614816

[B18] Funnell D. L. Lawrence C. B. Pedersen J. F. Schardl C. L. (2004). Expression of the tobacco β-1,3-glucanase gene, PR-2d, following induction of SAR with *Peronospora tabacina* . Physiol. Mol. Plant P 65, 285–296. doi: 10.1016/j.pmpp.2005.02.010

[B19] García-Olmedo F. Molina A. Alamillo J. M. Rodríguez-Palenzuéla P. (1998). Plant defense peptides. Pept. Sci. 47, 479–491. doi: 10.1002/(sici)1097-0282(1998)47:6<479::aid-bip6>3.0.co;2-k 10333739

[B20] Gharbi Y. Barkallah M. Bouazizi E. Hibar K. Gdoura R. Triki M. A. (2017). Lignification, phenols accumulation, induction of PR proteins and antioxidant-related enzymes are key factors in the resistance of *Olea europaea* to verticillium wilt of olive. Acta Physiol. Plant 39, 43. doi: 10.1007/s11738-016-2343-z

[B21] Giovannetti M. Mosse B. (1980). An evaluation of techniques for measuring vesicular arbuscular mycorrhizal infection in roots. New Phytol. 84, 489–500. doi: 10.1111/j.1469-8137.1980.tb04556.x

[B22] Gong M. Tang M. Chen H. Zhang Q. Feng X. (2013). Effects of two *Glomus* species on the growth and physiological performance of *Sophora davidii* seedlings under water stress. New For. 44, 399–408. doi: 10.1007/s11056-012-9349-1

[B23] Gregor J. Maršálek B. (2004). Freshwater phytoplankton quantification by chlorophyll a: a comparative study of *in vitro*, *in vivo* and *in situ* methods. Water Res. 38, 517–522. doi: 10.1016/j.watres.2003.10.033 14723919

[B24] Guo J. Ma X. Cai Y. Ma Y. Zhan Z. Zhou Y. J.. (2016). Cytochrome P450 promiscuity leads to a bifurcating biosynthetic pathway for tanshinones. New Phytol. 210, 525–534. doi: 10.1111/nph.13790 26682704 PMC4930649

[B25] Gupta P. Hirschberg J. (2022). The genetic components of a natural color palette: A comprehensive list of carotenoid pathway mutations in plants. Front. Plant Sci. 12. doi: 10.3389/fpls.2021.806184 PMC8770946 35069664

[B26] Gutjahr C. Casieri L. Paszkowski U. (2009). *Glomus intraradices* induces changes in root system architecture of rice independently of common symbiosis signaling. New Phytol. 182, 829–837. doi: 10.1111/j.1469-8137.2009.02839.x 19383099

[B27] Hammad A. M. M. El-Mohandes M. A. O.. (1999). Controlling fusarium wilt disease of cucumber plants via antagonistic microorganisms in free and immobilized states. Microbiol Res. 154, 113–117. doi: 10.1016/s0944-5013(99)80002-0

[B28] Hanks J. N. Snyder A. K. Graham M. A. Shah R. K. Blaylock L. A. Harrison M. J.. (2005). Defensin gene family in *Medicago truncatula*: structure, expression and induction by signal molecules. Plant Mol. Biol. 58, 385–399. doi: 10.1007/s11103-005-5567-7 16021402

[B29] Hura K. Hura T. Dziurka K. Dziurka M. (2014). Biochemical defense mechanisms induced in winter oilseed rape seedlings with different susceptibility to infection with *Leptosphaeria maculans* . Physiol. Mol. Plant P 87, 42–50. doi: 10.1016/j.pmpp.2014.06.001

[B30] Ismail Y. Hijri M. (2012). Arbuscular mycorrhisation with *Glomus irregulare* induces expression of potato PR homologues genes in response to infection by *Fusarium sambucinum* . Funct. Plant Biol. 39, 236–245. doi: 10.1071/fp11218 32480777

[B31] Jain S. Choudhary D. K. (2014). Induced defense-related proteins in soybean (*Glycine max* l. Merrill) plants by *Carnobacterium* sp. SJ-5 upon challenge inoculation of *Fusarium oxysporum* . Planta 239, 1027–1040. doi: 10.1007/s00425-014-2032-3 24504695

[B32] Jung S. C. Martinez-Medina A. Lopez-Raez J. A. Pozo M. J. (2012). Mycorrhiza-induced resistance and priming of plant defenses. J. Chem. Ecol. 38, 651–664. doi: 10.1007/s10886-012-0134-6 22623151

[B33] Kumar M. Brar A. Yadav M. Chawade A. Vivekanand V. Pareek N. (2018). Chitinases–potential candidates for enhanced plant resistance towards fungal pathogens. Agriculture-london 8, 88. doi: 10.3390/agriculture8070088

[B34] Li B. Cui G. Shen G. Zhan Z. Huang L. Chen J.. (2017). Targeted mutagenesis in the medicinal plant *Salvia miltiorrhiza* . Sci. Rep-uk 7, 43320. doi: 10.1038/srep43320 PMC5335714 28256553

[B35] Li Y. Duan T. Nan Z. Li Y. (2021). Arbuscular mycorrhizal fungus alleviates alfalfa leaf spots caused by *Phoma medicaginis* revealed by RNA-seq analysis. J. Appl. Microbiol. 130, 547–560. doi: 10.1111/jam.14387 31310670

[B36] Liu L. Yang D. Xing B. Zhang H. Liang Z. (2018). *Salvia castanea* hairy roots are more tolerant to phosphate deficiency than *Salvia miltiorrhiza* hairy roots based on the secondary metabolism and antioxidant defenses. Mol. J. Synthetic Chem. Nat. Prod. Chem. 23, 1132. doi: 10.3390/molecules23051132 PMC6099837 29747474

[B37] Lobato A. Gonçalves-Vidigal M. Filho P. V. Andrade C. Kvitschal M. Bonato C. (2010). Relationships between leaf pigments and photosynthesis in common bean plants infected by anthracnose. New Zeal J. Crop Hort 38, 29–37. doi: 10.1080/01140671003619308

[B38] Mitsuhara I. Iwai T. Seo S. Yanagawa Y. Kawahigasi H. Hirose S.. (2008). Characteristic expression of twelve rice PR1 family genes in response to pathogen infection, wounding, and defense-related signal compounds (121/180). Mol. Genet. Genomics 279, 415–427. doi: 10.1007/s00438-008-0322-9 18247056 PMC2270915

[B39] Mozzetti C. Ferraris L. Tamietti G. Matta A. (1995). Variation in enzyme activities in leaves and cell suspensions as markers of incompatibility in different phytophthora-pepper interactions. Physiol. Mol. Plant P 46, 95–107. doi: 10.1006/pmpp.1995.1008

[B40] Mustafa G. Khong N. G. Tisserant B. Randoux B. Fontaine J. Magnin-Robert M.. (2017). Defence mechanisms associated with mycorrhiza-induced resistance in wheat against powdery mildew. Funct. Plant Biol. 44, 443–454. doi: 10.1071/fp16206 32480577

[B41] Nair A. Kolet S. P. Thulasiram H. V. Bhargava S. (2015). Systemic jasmonic acid modulation in mycorrhizal tomato plants and its role in induced resistance against *Alternaria alternata* . Plant Biol. 17, 625–631. doi: 10.1111/plb.12277 25327848

[B42] Neeraj Singh K. (2011). Organic amendments to soil inoculated arbuscular mycorrhizal fungi and *Pseudomonas fluorescens* treatments reduce the development of root-rot disease and enhance the yield of *Phaseolus vulgaris* l. Eur. J. Soil Biol. 47, 288–295. doi: 10.1016/j.ejsobi.2011.07.002

[B43] Pan S. Q. (1991). A technique for detection of chitinase, β -1,3-Glucanase, and protein patterns after a single separation using polyacrylamide gel electrophoresis or isoelectrofocusing. Phytopathology 81, 970. doi: 10.1094/phyto-81-970

[B44] Paszkowski U. (2006). A journey through signaling in arbuscular mycorrhizal symbioses 2006. New Phytol. 172, 35–46. doi: 10.1111/j.1469-8137.2006.01840.x 16945087

[B45] Pečenková T. Pejchar P. Moravec T. Drs M. Haluška S. Šantrůček J.. (2022). Immunity functions of arabidopsis pathogenesis-related 1 are coupled but not confined to its c-terminus processing and trafficking. Mol. Plant Pathol. 23, 664–678. doi: 10.1111/mpp.13187 35122385 PMC8995067

[B46] Phillips J. M. Hayman D. S. (1970). Improved procedures for clearing roots and staining parasitic and vesicular-arbuscular mycorrhizal fungi for rapid assessment of infection. T. Brit. Mycol. Soc. 55, 158–IN18. doi: 10.1016/s0007-1536(70)80110-3

[B47] Qi D. Zou L. Zhou D. Zhang M. Wei Y. Li K.. (2022). Biocontrol potential and antifungal mechanism of a novel *Streptomyces sichuanensis* against *Fusarium oxysporum* f. sp. cubense tropical race 4 *in vitro* and *in vivo* . Appl. Microbiol. Biot. 106, 1633–1649. doi: 10.1007/s00253-022-11788-3 35141868

[B48] Ritchie R. J. Bunthawin S. (2010). The use of pulse amplitude modulation (PAM) fluorometry to measure photosynthesis in a CAM orchid, *Dendrobium* spp. (D. cv. viravuth pink). Int. J. Plant Sci. 171, 575–585. doi: 10.1086/653131

[B49] Robert-Seilaniantz A. Grant M. Jones J. D. G. (2011). Hormone crosstalk in plant disease and defense: More than just JASMONATE-SALICYLATE antagonism. Annu. Rev. Phytopathol. 49, 317–343. doi: 10.1146/annurev-phyto-073009-114447 21663438

[B50] Serrano I. Audran C. Rivas S. (2016). Chloroplasts at work during plant innate immunity. J. Exp. Bot. 67, 3845–3854. doi: 10.1093/jxb/erw088 26994477

[B51] Shi M. Huang F. Deng C. Wang Y. Kai G. (2018). Bioactivities, biosynthesis and biotechnological production of phenolic acids in *Salvia miltiorrhiza* . Crit. Rev. Food Sci. 59, 1–40. doi: 10.1080/10408398.2018.1474170 29746788

[B52] Shine M. B. Yang J. El-Habbak M. Nagyabhyru P. Fu D. Navarre D.. (2016). Cooperative functioning between phenylalanine ammonia lyase and isochorismate synthase activities contributes to salicylic acid biosynthesis in soybean. New Phytol. 212, 627–636. doi: 10.1111/nph.14078 27411159

[B53] Song Y. Chen D. Lu K. Sun Z. Zeng R. (2015). Enhanced tomato disease resistance primed by arbuscular mycorrhizal fungus. Front. Plant Sci. 6. doi: 10.3389/fpls.2015.00786 PMC4585261 26442091

[B54] Soudani S. Poza-Carrión C. Gómez N. D. González-Coloma A. Andrés M. F. Berrocal-Lobo M. (2022). Essential oils prime epigenetic and metabolomic changes in tomato defense against *Fusarium oxysporum* . Front. Plant Sci. 13. doi: 10.3389/fpls.2022.804104 PMC9002333 35422834

[B55] Sowik I. Borkowska B. Markiewicz M. (2016). The activity of mycorrhizal symbiosis in suppressing verticillium wilt in susceptible and tolerant strawberry (Fragaria x ananassa duch.) genotypes. Appl. Soil Ecol. 101, 152–164. doi: 10.1016/j.apsoil.2016.01.021

[B56] Tada Y. Spoel S. H. Pajerowska-Mukhtar K. Mou Z. Song J. Wang C.. (2008). Plant immunity requires conformational charges of NPR1 *via* s-nitrosylation and thioredoxins. Science 321, 952–956. doi: 10.1126/science.1156970 18635760 PMC3833675

[B57] Tian S. Torres R. Ballester A. R. Li B. Vilanova L. González-Candelas L. (2016). Molecular aspects in pathogen-fruit interactions: Virulence and resistance. Postharvest Biol. Tec 122, 11–21. doi: 10.1016/j.postharvbio.2016.04.018

[B58] Vos C. Schouteden N. Tuinen D. Chatagnier O. Elsen A. Waele D. D.. (2013). Mycorrhiza-induced resistance against the root–knot nematode meloidogyne incognita involves priming of defense gene responses in tomato. Soil Biol. Biochem. 60, 45–54. doi: 10.1016/j.soilbio.2013.01.013

[B59] Wang R. Wang G. L. Ning Y. (2019). PALs: Emerging key players in broad-spectrum disease resistance. Trends Plant Sci. 24, 785–787. doi: 10.1016/j.tplants.2019.06.012 31300196

[B60] Wang Y. Yin Q. Qu Y. Li G. Hao L. (2018). Arbuscular mycorrhiza-mediated resistance in tomato against *Cladosporium fulvum*-induced mould disease. J. Phytopathol. 166, 67–74. doi: 10.1111/jph.12662

[B61] Wang Y. Zhang S. Yin X. Liu J. Wu F. (2016). Isolation and identification of arbuscular mycorrhizal fungi from mainland China. Microbiol. China 10, 2154–2165. doi: 10.13344/j.microbiol.china.150921

[B62] Xu X. Chen Y. Li B. Zhang Z. Qin G. Chen T.. (2022). Molecular mechanisms underlying multi-level defense responses of horticultural crops to fungal pathogens. Hortic. Res. 9, uhac066. doi: 10.1093/hr/uhac066 35591926 PMC9113409

[B63] Yang L. Miao Z. Yang G. Shao A. Huang L. Shen Y.. (2013). Fusarium wilt of *Salvia miltiorrhiza* and its pathogenic bacteria. China J. Chin. Material Med. 38, 4040–4043. doi: 10.4268/cjcmm20132309 24791484

[B64] Yen P. P. Pratap-Singh A. (2021). Vacuum microwave dehydration decreases volatile concentration and soluble protein content of pea (*Pisum sativum* l.) protein. J. Sci. Food Agr. 101, 167–178. doi: 10.1002/jsfa.10627 32613616

[B65] Zai X. M. Zhu S. N. Qin P. Wang X. Y. Che L. Luo F. X. (2012). Effect of *Glomus mosseae* on chlorophyll content, chlorophyll fluorescence parameters, and chloroplast ultrastructure of beach plum (*Prunus maritima*) under NaCl stress. Photosynthetica 50, 323–328. doi: 10.1007/s11099-012-0035-5

[B66] Zhang H. Jin W. Zhu X. Liu L. He Z. Yang S.. (2016). Identification and characterization of *Salvia miltiorrhiza* in miRNAs in response to replanting disease. PLoS One 11, e0159905. doi: 10.1371/journal.pone.0159905 27483013 PMC4970794

[B67] Ziv C. Zhao Z. Gao Y. G. Xia Y. (2018). Multifunctional roles of plant cuticle during plant-pathogen interactions. Front. Plant Sci. 9. doi: 10.3389/fpls.2018.01088 PMC6068277 30090108

