# Supplementary material for: Arbuscular mycorrhizal fungi enhance disease resistance of Salvia miltiorrhiza to Fusarium wilt
Source: Front Plant Sci. 2022 Dec 1;13:975558. doi: 10.3389/fpls.2022.975558 (PMC9753693; doi:10.3389/fpls.2022.975558)

Figure S1 Photomicrographs of *G. versiforme*.


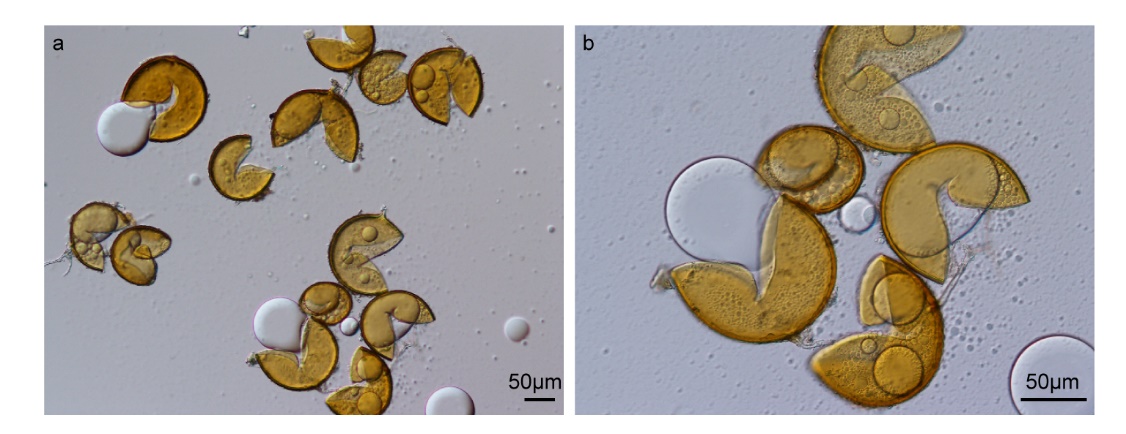


Supplemental Table 1 Genes and primers used in quantitative real-time PCR analysis.

| Gene | Forward primer (5’-3’) | Reverse primer (5’-3’) | Amplification efficiency (%) |
| --- | --- | --- | --- |
| *SmLOX* | CCATTTATTATCGCCACCAAC | TGTCATCTCAACAAACCCTCC | 91.563 |
| *SmAOS* | CACGCCACCGTCAACAAC | CGATGCCGCAACCTCAATAT | 98.632 |
| *SmAOC* | CAACTCCATCCAAGGTTCAGG | TCGCCGGAGTAGAGCTTGTTG | 100.319 |
| *SmOPR* | GTCGAGAGACTGAAGCTCAAC | CAGCATTAGATTGCAGGAAG | 95.785 |
| *SmJAR* | AAGTTTGTGCCTTTCAACGACG | GGAAATCTGGTCCGAAGATGACT | 93.628 |
| *SmPDF2.1* | TGAGCAGCTTTTTGAGGTTGTTTG | GAGTAGATTTAGTTAACAGTGTCTGG | 92.507 |
| *SmPAL* | GCCAGCAGCGATTGGGTTATG | GGAGTAGCCTTGGAGGAGGGTGT | 97.788 |
| *SmNPR1* | AGGAAGGGAAGCCGAGGTATTA | CAGGCATCGTGAGCACATAAACTA | 91.833 |
| *SmPR1* | GGTGGAGTTGTGGGTGAATGA | TAAGGACGTTGGCCGATGTAG | 105.705 |
| *SmPR10* | CCATTTCTCCAAATTCCAAGAG | CCTCGTCTCTTTCAGTAGTC | 102.805 |
| *SmActin* | GGTGCCCTGAGGTCCTGTT | AGGAACCACCGATCCAGACA | 101.097 |

Figure S2 qPCR standard curve of defense-related genes.


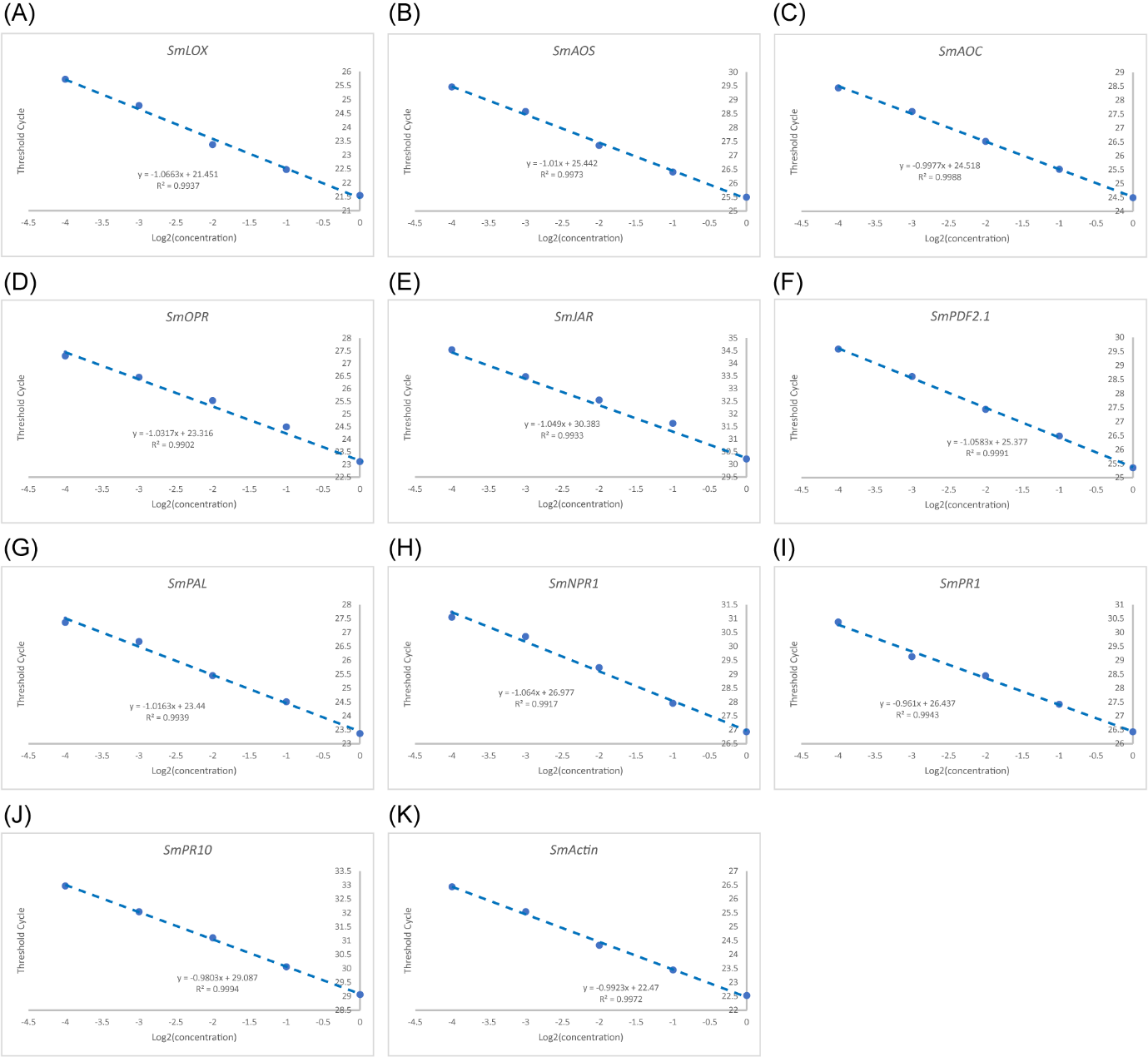

Supplement: Supplementary file 1 [file DataSheet_1.docx]
